# Effects of the Physiological Status and Diet on Blood Metabolic Parameters in Amiata Dairy Donkeys

**DOI:** 10.3390/ani11113292

**Published:** 2021-11-17

**Authors:** Federica Salari, Cristina Roncoroni, Iolanda Altomonte, Carlo Boselli, Giovanni Brajon, Mina Martini

**Affiliations:** 1 Department of Veterinary Science, University of Pisa, 56124 Pisa, Italy; federica.salari@unipi.it (F.S.); mina.martini@unipi.it (M.M.); 2 Istituto Zooprofilattico Sperimentale Lazio e Toscana, 00178 Roma, Italy; cristina.roncoroni@izslt.it (C.R.); carlo.boselli@izslt.it (C.B.); 3 Interdepartmental Centre for Research in Agricultural and Environmental Sciences “Enrico Avanzi”, University of Pisa, 56122 Pisa, Italy; 4 Istituto Zooprofilattico Sperimentale del Lazio e della Toscana M. Aleandri, 50018 Florence, Italy; giovanni.brajon@izslt.it; 5 Interdepartmental Research Center Nutraceuticals and Food for Health, University of Pisa, 56124 Pisa, Italy

**Keywords:** jenny, donkey feeding, pregnancy, lactation, blood urea nitrogen, NEFA, B-HBA, milk quality, milk fatty acid, somatic cell count

## Abstract

**Simple Summary:**

This study investigated the physiological responses of donkeys feeding two different diets during maintenance, pregnancy and lactation. To investigate how the metabolic state influences dairy production, we also assessed the relationships between the blood metabolic profile and milk quality. We found that pregnancy and the first months of lactation led to lower feed intake and increases in blood non-esterified fatty acids (NEFAs), which was linked to the difficulty that the jennies have in meeting the higher energy needs. The intake of the concentrate in the diet was associated with the increase in blood glucose, both during maintenance and lactation. Higher NEFA were related to lower de novo synthesized milk fatty acids, whereas higher plasma B-HBA were related to higher somatic cell count. This paper contributes to the knowledge of physiological responses of jennies during milk production phases and provides information for donkey milk producers.

**Abstract:**

Body weight changes and blood metabolic parameters in jennies feeding two different diets and in three physiological statuses were investigated (maintenance vs. pregnancy; maintenance vs. lactation). The relationships between blood metabolic profile and milk quality were also evaluated. Fourteen jennies were allocated to two groups (1: pregnant/lactating; 2: non-pregnant, non–lactating). Pregnant jennies and maintenance jennies (during the first 10-week measurement period) fed a diet consisted of ad libitum grass hay (diet 1); lactating jennies and maintenance jennies (during the last 10-week measurement period) fed ad libitum grass hay plus 2 kg/head/day of concentrate (diet 2). Blood sampling was performed on the jennies of both groups; individual milk samples were also collected during the first 70 days in milk. Higher blood NEFA (*p* < 0.05) were found in pregnant compared to maintenance jennies (diet 1) (68 vs. 37 μmol/L). Lactating jennies showed higher (*p* < 0.01) average blood NEFA (268 vs. 26 μmol/L) and glucose (66 vs. 55 mg/dL) compared to the maintenance (diet 2). Blood glucose was positively correlated to milk fat (*p* < 0.05), while negative significant correlations between de novo milk fatty acids and NEFAs were observed. Positive correlations between plasma B-HBA and somatic cell count (*p* < 0.01) were also found.

## 1. Introduction

In industrialized countries donkeys provide different roles (i.e., donkey-assisted activities for humans) and also produce meat and milk. Donkeys represent an effective source of biodiversity and have links with the rural/local economy. On the other hand, in poor communities, equids have working roles such as the transport of materials for sale and the harvesting, transport and sale of crops. Donkeys provide transportation, income generation, empowerment and food security to marginalized groups and families.

Donkey milk is similar in composition to human milk and has shown beneficial properties in vivo and in vitro studies [1]. The number of farms breeding donkey for milk production is increasing, especially in Europe and in Asia. Donkey farming has moved from traditional forms of management to more organized, intensive and semi-intensive farming systems. These changes in donkey farming mean that specific knowledge of the milk production, nutrition and welfare of donkeys is required [2,3].

Feed guidelines are available above all for resting and working donkeys, rather than those involved in milk production [4].

Pregnancy and lactation are physiological phases that result in increased metabolic demands. In addition, knowledge of the nutritional status in the various physiological phases is particularly important as a result of the increased metabolic demands during pregnancy and lactation, which involve the energy metabolism of carbohydrates and fats.

The lean body mass loss during lactation may also cause protein mobilization from the skeletal muscle to provide amino acid precursors for milk and energy for gluconeogenesis required for the synthesis of lactose [5]. Similarly, in horses, the final weeks pre-partum and the beginning of lactation result in a mild catabolic state [6].

Therefore, during pregnancy and lactation, body modifications and changes in different blood metabolic parameters can occur and are often indicative of changes in an animal’s physiological state, such as energy demands and health condition.

If pregnancy and lactation are not carefully managed, jennies may be exposed to metabolic disorders such as laminitis, equine metabolic syndrome and hyperlipidemia [7].

Pregnancy and lactation are important drivers for milk production level and economic performance. In dairy cows, poor feeding, low or high body condition score at calving and metabolic diseases in early lactation negatively affect milk production [8].

Body weight, feed ingestion and blood metabolic parameters measurements are important to evaluate donkey health [9] and productive performance. Furthermore, fewer investigations have focused on the physiological phases [10,11] and diet in dairy jennies compared to other livestock species, and to the best of our knowledge no reference values are available for biochemical parameters in pregnancy and lactation.

The aim of this study was thus to evaluate the donkeys’ physiological responses in terms of body condition score and weight, feed ingestion and blood metabolic parameters in jennies in three different physiological statuses (maintenance vs. pregnancy; maintenance vs. lactation) feeding two different diets.

In addition, in order to understand how the metabolic condition affects milk production, we investigated the relationships of the blood metabolic profile with milk production and quality.

## 2. Materials and Methods

### 2.1. Animal Management, Experimental Diets and Samplings

The experiment was carried out between June and November 2018. Fourteen healthy multiparous Amiata jennies (BW 316 ± 29 kg between 6 to 18 years) were enrolled in the study. These jennies were located in a farm in Tuscany (central Italy) and were reared in a semi-intensive system for the production of milk intended for human consumption, in compliance with Regulation (EC) No. 853/2004.

At the beginning of the studies donkeys were weighted using a scale and physically separated into two groups with similar average body weight and age. They were managed in two different paddocks as follows: seven jennies (group 1: pregnant/lactating group) and seven jennies (group 2: non-pregnant, non-lactating). The feeding period for the pregnant/lactating group included 10 weeks prior to parturition and 10 weeks postpartum. Foals and lactating jennies were managed in the same paddock. The non-pregnant jennies in maintenance were managed over the same period, i.e., for 20 weeks.

The groups were fed two different diets. Diet 1 consisted of ad libitum grass hay, which was fed to the jennies in group 1 during pregnancy and to the group 2 during the first 10-week measurement period (from Week 1 to Week 10). Diet 2 consisted of the same ad libitum grass hay used in Diet 1 supplemented with 2 kg/head/day of commercial concentrate. Diet 2 was fed to group 1 during lactation and to group 2 during an additional 10-week measurement period (from Week 11 to Week 20). To achieve a target refusal rate of 10%, any refusal of grass hay was collected each day in the morning before fresh grass hay was given, and the amount of feed offered was adjusted daily based on the intake of the previous day The feeds offered and refused were weighed daily in order to calculate the daily voluntary intake for each group. Animals had free access to clean fresh water.

In both groups there was a transition period from Diet 1 to Diet 2. The transition lasted eight days and consisted in a gradual increase of concentrate (+250 g per day). In particular, attention was paid to pregnant and lactating donkeys that are predisposed to hyperlipidemia [4].

After parturition, milk samples were collected from the jennies in group 1 at Days 14, 28, 42, 56 and 70 post-partum, with a total of 35 individual milk samples. During the milk sampling days, jennies were machine-milked twice (at 11.00 and 17:00). The foals were separated from their mothers three hours before each milking, and the milk yield was recorded using a lactometer connected to the milking machine. After collection, milk samples were refrigerated at 4 °C, and then analyzed for gross chemical composition within 24 h. Body weight (BW) was measured once a week (the same day starting from 7:00 a.m.) in donkeys from both groups using a scale, and body condition score (BCS) was assessed through a score from 1 to 5, following the system developed by the Donkey Sanctuary [4].

### 2.2. Chemical Analysis of Hay, and Estimate of the Energy and Protein Value of Feed

Daily samples of hay were taken and pooled to form a single composite sample from which representative subsamples (three replicates) were analyzed in terms of chemical composition following the Association of Official Analytical Chemists [12]. The chemical composition of the concentrate was obtained from the manufacturer (Mignini and Petrini SpA).

Throughout the study, hay and concentrate belonging to the same batch were composed of the following ingredients: wheat bran, alfalfa flour, dried beet pulp, corn, decorticated sunflower seed flour, barley, decorticated soya seeds flour, horse bean, distillers’ and soluble dried corn, soya seed hulls, soluble condensed molasses, calcium carbonate, corn gluten flour, sugar cane molasses, sodium bicarbonate, sodium chloride, dicalcium phosphate and vitamin and mineral supplements. The vitamin and mineral contents of the commercial feed (per kg of feed) was: Vit. A 20,400 U.I.; Vit. D3 2720 U.I.; Vit. E 30.6 mg; cobalt carbonate II in coated granules 0.41 mg; calcium iodate anhydrous in coated granules 2.09 mg; manganese oxide II 87.72 mg; sodium selenite 0.45 mg; and zinc oxide 84.32 mg. The chemical composition and nutritive value of hay and concentrate for the donkeys’ diet is reported in Table 1.

### 2.3. Blood Analysis

Blood sampling was performed on jennies from both groups at the end of pregnancy and at Days 14, 28, 42, 56 and 70 post-partum. Blood samples were collected from the jugular vein by vacuum collection clot activator tubes (Vacutest plast; Vacutest Kima, Padua, Italy). Serum samples were frozen at −20 °C and stored under these conditions until analyzed. Blood urea nitrogen (BUN), non-esterified fatty acids (NEFAs), glucose, beta-hydroxybutyrate (B-HBA) and total protein (TP) serum levels were analyzed using an automatic biochemical analyzer (Olympus 4000; Beckman Coulter, Milan, Italy).

### 2.4. Analysis of Milk Chemical Composition and Somatic Cell Count

The following parameters were evaluated in the donkey milk samples: dry matter (DM) and ash using the methods of the Association of Official Analytical Chemists [12], proteins, caseins, fat and lactose contents by infrared analysis (MilkoScanTM 7RM; Italian Foss Electric, Padua, Italy) and pH using the potentiometric method. Somatic cell count (SCC) was performed by fluoro-optoelectronic microscopy (Fossomatic 90; Foss, Hileroed, Denmark).

Fatty acids were evaluated as described by Martini et al. [13].

### 2.5. Statistical Analysis

Data milk and blood metabolic parameters were evaluated using Shapiro–Wilk Test and the Kolmogorov-Smirnov test respectively to verify the normality of the errors; Bartlett’s tests was used to verify the homogeneity of variances. The variables that did not satisfy the ANOVA assumptions were submitted to the square root transformation [y ^′ = √ (y + 0.5)].

Weight, BCS and blood parameters of the donkeys fed with Diet 1 were analyzed using a model for repeated measurements with the physiological phase (maintenance vs. pregnancy) as a fixed effect, and the subject as a random effect.

Weight, BCS and blood parameters of the donkeys fed with Diet 2 were analyzed using a model for repeated measurements with the physiological phase (maintenance vs. lactation) and the day of sampling as fixed effects, and the subject as a random effect.

In addition, Pearson correlations were calculated between weight, BCS, blood parameters, milk quality, and the acid profile of the milk of donkeys in the first 70 days of lactation.

Least significance means were compared by Tukey’s test. Significant differences were considered at *p* < 0.05. Statistical analysis was carried out using SAS [14].

## 3. Results and Discussion

The pregnant and lactating groups tended to have lower feed intakes compared to the maintenance groups fed the same diet (Table 2). The lowest intakes were in pregnant jennies, which is probably due to the lower abdominal space available for feed [4] because of the abdominal cavity occupied by the conceptus, membranes and fluids.

In the maintenance jennies the feed intake was comparable with Pearson’s findings (2–2.7 kg/100 kg of BW; corresponding to about 7.2–8.5 kg per day on wet basis) [15]. The feed ingestion in our lactating donkeys was quite similar to those described by Fantuz et al. [16] in Martina Franca lactating jennies (2.8–3.2 kg/100 kg BW; corresponding to about 8.84–10.11 kg per day on wet basis). Considering the ingestion levels in the different groups (Table 2), energy requirements were likely reached for pregnant (diet 1) and lactating jennies (diet 2), while with both diets’ maintenance donkeys exceeded the energy requirements present in the literature. In fact, for sedentary donkeys that are not pregnant, lactating or growing, the daily maintenance energy requirements are approximately 13 MJ DE/100 kg BW [17]. In mid- to late-pregnancy, approximately 20–30% of the energy requirements should be added; while lactation seems to require approximately twice as much energy as in maintenance [17].

No significant differences in BCS were found between the two groups (pregnant vs. maintenance) fed the same diet (diet 1), and the weight differences between the groups refer to the fetus and fetal membranes weight (Table 3).

There were no significant differences in blood metabolic parameters between the maintenance and pregnant groups fed the same diet (diet 1), except for the NEFAs which showed a significant increase (*p* < 0.05) in the pregnant group.

NEFAs are a biomarker of fat metabolism and in equines increase after exercise or due to anorexia or food restrictions [18]. Increases in NEFAs may be adaptive evolution strategy to face under harsh conditions, enabling donkeys to maintain glucose levels within the normal range [19]. Changes in NEFAs in donkeys have also been found in relation to diet [5], starving, age, sex and physiological phase [20].

NEFA concentrations in the pregnant and maintenance jennies fed diet 1 (Table 3) were comparable to the horse in the postprandial period after hay ingestion (about 50 μmol/L) [21] and below the reference range reported for ponies (100–500 μmol/L; Watson et al., 1992 cited by Brinkmann et al. [19]. Moreover, in our study, NEFA levels in the pregnant jennies were below the values obtained by Chiofalo et al. [20] in pregnant Pantesco donkeys (120.21 μmol/L); this difference is likely due to an unbalanced forage/concentrate ratio of the diet and to a negative energetic balance in the study of Chiofalo et al. [20] as also highlighted by the low BCS (mean 2.5).

The significant increase (*p* < 0.01) in NEFAs that we found during pregnancy compared to maintenance, could be related to the decreased ingestion that normally occurs in the last phases of pregnancy, which leads to lipomobilization.

In maintenance and pregnant animals fed only hay (Diet 1), the B-HBA levels were quite similar to those described in non-energy restricted Shetland pony mares (2.6–4.68 mg/dL) [19]. This may indicate that B-HBAs were in the normal range and the jennies were not suffering from ketosis.

Excessive increases in B-HBA are indicative of ketosis, since a few circulating NEFAs are used as an energy source by tissues, while the rest is transported to the liver and converted into ketone bodies (e.g., B-HBA, acetone), which can partly replace glucose in the tissues. However, in equids the disposition for ketone body generation is limited [19].

In our study, the two groups of animals (maintenance and pregnancy, Diet 1) did not show significant differences in blood glucose levels. Blood glucose was slightly lower than the average glucose found in the Martina Franca breed and in the Brazilian donkey (58–59 mg/dL) [22,23]. The greatest differences compared to the literature were observed for the group of pregnant jennies which had lower glucose values than those reported in pregnancy for Martina Franca (ranging between 73 and 78 mg/dL) [24] and Amiata donkeys (ranging between 78 and 91 mg/dL) [25].

However, the higher glucose values of pregnant jennies found by Bonelli et al. and Gloria et al. [24,25] could be linked to nutrition, as their diet for the jennies was integrated with commercial concentrate for equines.

According to Firshman and Valberg [26], although resting glucose and insulin concentrations are similar in pregnant and non-pregnant mares, pregnancy together with obesity are risk factors for developing insulin resistance in equids.

TP and BUN are biomarkers of protein metabolism and provide information about the nutritional status of animals. Protein requirements were covered in all groups and in both diets, with the exception of the pregnant group in which they were slightly lower (Table 2).

The TP values in the different groups (Table 2) were similar to the reference parameters reported by Burden et al. [27] for maintenance donkeys ranging from 5.8–7.6 mg/dL. TP for donkeys in maintenance were also similar to Dezzutto et al. [28] and Caldin et al. [29] (7.3 and 6.9 mg/dL, respectively) for adult and pregnant donkeys, and are in agreement with the studies on the same breed in the same physiological phase (7.62, 7.87 mg/dL) [10,25].

BUN serum values were quite similar to the mean value reported for adult donkeys (16 mg/dL) [23], and to the average levels found in Martina Franca jennies in late pregnancy (18–20 mg/dL) [24], while higher mean values were observed in Ragusana jennies (27 mg/dL) [29]. In agreement with other studies on donkeys [24] and on several horse breeds [30], in our study there were no significant changes in either TP or BUN in pregnant compared to maintenance donkeys (Diet 1).

The BCS was lower (*p* < 0.01) in the lactating than in the maintenance animals, both of whose diets were supplemented with concentrate (Diet 2) (Table 4). The lactating jennies had quite a good BCS (3), but the maintenance group became overweight as the diet with the addition of concentrate exceeded the energy needs (Table 4).

There were more significant increases in glucose (*p* < 0.01) in the lactating donkeys compared to the maintenance group, probably due to the physiological phase (Table 4). However, glucose remained quite low compared to values reported for lactating donkeys in the same breed (78.5–98-mg/dL) [25].

The glucose supply for milk synthesis involves insulin action and the somatotropic axis. During early lactation insulin sensitivity is low, and the growth hormone (GH)-IGF-I axis is uncoupled to favor the mobilization of body energy reserves and the provision of substrates for milk production, such as glucose [31].

NEFAs also increased significantly (*p* < 0.01) tenfold in lactating animals compared to the maintenance group following the same diet. This change could be due to the negative balance in early lactation due to decreased appetite and increased needs required for milk production. In any case, the NEFA values fell within the reference range reported for ponies (100–500 μmol/L; Brinkmann et al. [19]).

Lactating jennies registered lower mean TP and BUN values than described by Dezzutto et al. [28] for early lactation jennies (8.3 and 25 mg/dL respectively).

**Figure 1:**
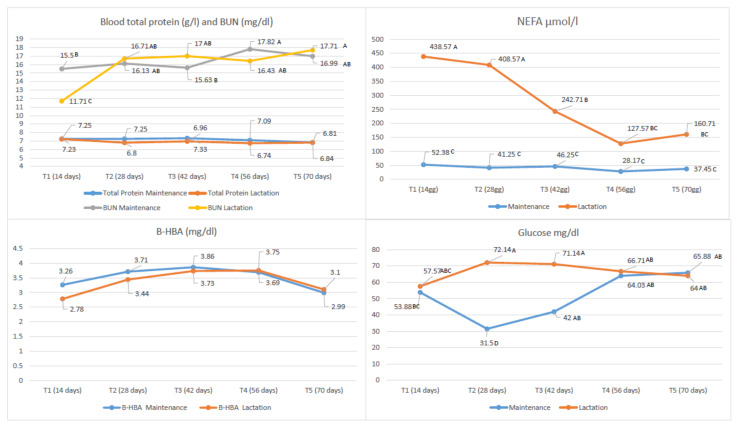
Trend of the blood metabolic parameters evaluated in the lactation and maintenance jennies fed with Diet 2. A,B,C,D = *p* < 0.01 Abbreviations: BUN: blood urea nitrogen; NEFA = Non Esterified Fatty Acid; B-HBA: Beta-Hydroxybutyrate.

The trend of the blood metabolic parameters during Diet 2 (Figure 1) revealed a peak in NEFAs at 14 days of lactation, they then remained significantly high at the beginning of lactation/diet compared to maintenance, and slowly decreased in time with lactation up to 42 days.

After 56–70 days of lactation/diet, the NEFAs became similar to the maintenance group and were comparable to the values reported by Fantuz et al. [16] (150–170 μmol/L) in jennies at approximately three months of lactation which were fed a digestible energy (DE, according to the NRC system) diet comparable with our study (about 22–24 DE/100 kg of body weight calculated).

Birth produces a rise in free fatty acids in both the maternal and fetal or neonatal circulation in most species, presumably because of the catecholamine-induced release of fatty acids from fat stores [32]. The decrease in NEFAs is most likely due to an improvement in the energy balance as lactation progresses.

The B-HBA values, on the other hand, had a stable trend over time, overlapping in the two groups.

Although on the average glucose was higher in lactation than in maintenance (*p* < 0.01; 66.52 and 55.43 mg/dL respectively) (Table 4), it was stable as lactation progressed. Donkeys in maintenance showed a different trend: at 42 days of diet from the introduction of the concentrate glycaemia significantly increased and settled towards values similar to those of lactating donkeys. The concentrate seems to have influenced the increase in blood sugar, although to different extents in the two groups.

The TP did not show significant differences between lactating and maintenance jennies (group 2) over the course of the diet. BUN was quite stable in maintenance donkeys, and showed minimum values in lactating donkeys at 14 days. BUN increased at 28 days in lactation (*p* < 0.01) settling towards levels similar to that of the maintenance group for the entire duration of the diet.

The increased levels of NEFAs and BUN would seem to indicate that in the first 28–56 days from foaling there are the highest protein/energy levels needs

As regards the correlations between blood metabolic parameters and milk quality (Table 5), glucose was positively correlated (*p* < 0.05) with the percentage of fat. This relationship was probably due to the fact that, although glucose is not a major precursor of fatty acid synthesis, glucose carbon is used to synthesize glycerol for triglyceride synthesis, as reported in cows [33].

We found that NEFAs had positive correlations (*p* < 0.05) with DM, protein, casein and ash; similarly positive correlations between NEFAs and milk protein percentage have been observed in cows [34]. Furthermore, in cows of high genetic merit milk protein secretion was significantly associated with higher NEFA concentrations [35]. Probably, the positive correlations between NEFAs and milk components are related to the energy needs for the synthesis of milk components, which therefore lead to increased lipomobilization.

Plasma B-HBA was positively (*p* < 0.01) correlated with milk SCC. B-HBA is a marker of ketonemia. Negative effects of hyperketonemia on immunity and positive correlations between ketosis and SCC have been observed in cows [35].

Blood TPs were positively (*p* < 0.05) correlated with milk casein and pH, while BUN (*p* < 0.05) was positively correlated with total milk protein content. Positive correlations between blood proteins, BUN and milk proteins have also been reported for Holstein dairy cows [36].

Several correlations between NEFAs and milk fatty acids were also found (Table 6): negative correlations with short-chain (SCFA) and saturated fatty acids (*p* < 0.01) and positive with long-chain (LCFA) and monounsaturated fatty acids (*p* < 0.01) were found. In particular, NEFAs showed negative correlations with SCFA (*p* < 0.05) and some medium chain (MCFA)-saturated fatty acids (C6:0, C8:0, C12:0, C17:0; *p* < 0.01; C10:0, C14:0; C15:0 *p* < 0.05) and with C20:0 (*p* < 0.05). In addition, NEFAs positively (*p* < 0.05) correlated with C16:1 and C24:0.

Similar correlations were also observed in dairy cows by Dórea et al. [37]. In cows the proportion of de novo milk fatty acids decreases when plasma NEFA concentration increases. SCFA and MCFA (C4:0–C14:0) are the result of de novo synthesis in the mammary gland. In fact, lower proportions of SCFAs and MCFAs in the milk during early lactation might be an indicator of body fat mobilization. Mobilization of adipose tissue might increase the supply of long-chain fatty acids (LCFAs), and the high uptake of LCFAs by the mammary gland inhibits de novo synthesis of SCFAs and MCFAs, causing changes in the composition of milk fatty acids [38].

## 4. Conclusions

In our study, pregnancy and the first months of lactation led to lower ingestion levels and increases in NEFAs, confirming the difficulty in these phases in meeting the greater energy needs similarly to other dairy species. The intake of concentrate in the diet contributes to the increase in blood sugar. Negative correlations between NEFAs and de novo milk fatty acids in milk are related to the energy balance: when lipomobilization increase the synthesis of milk fatty acids decrease. Finally, the correlations between plasma B-HBA and somatic cell count of milk could support negative effects of this parameter on the animal’s immune status.

## Figures and Tables

**Table 1:** Chemical composition and nutritive value of hay and concentrate for the donkeys’ diet. All components are expressed on a dry matter basis.

Components	Grass Hay	Commercial Concentrate
CP (g/100 g of DM)	8.53	18.97
Fat (g/100 g of DM)	1.05	3.45
Crude fiber (g/100 g of DM)	37.85	15.52
Ash (g/100 g of DM)	7.41	9.77
Sugars (g/100 g of DM)	n.d.	5.52
Starch (g/100 g of DM)	n.d.	23.56
NFC (g/100 g of DM)	19.39	35.40
Neutral Detergent Fiber (% of crude fiber)	63.62	32.41
Acid Detergent Fiber (% of crude fibre)	38.93	15.98
Acid Detergent Lignin (% of crude fiber)	6.91	3.45
Hemicellulose (% of crude fiber)	24.69	16.43
DE (MJ/kg DM)	8.42	11.56
DP (g/kg of DM)	68.37	182.99

Abbreviations: CP: crude protein; DM: dry matter; n.d: not determined; NFC: non-fibrous carbohydrate; DE: Digestible Energy (according to the NRC system); DP: Digestible crude protein.

**Table 2:** Average daily intake for group: Diet 1 and Diet 2.

	Diet 1		Diet 2	
	Pregnancy	Maintenance	Lactation	Maintenance
Hay (kg DM)	6.35	7.63	6.29	7.08
Concentrate (kg DM)	0	0	1.74	1.74
Total intake (kg DM)	6.35	7.63	8.03	8.82
Actual DE intake	14.86	21.60	25.58	25.28
(MJ/100 kg BW)				
Recommended DE intake ^a^	13–18	12–15	24–30	12–15
(MJ/100 kg BW)				
Actual CP intake	150.55	218.79	303.41	296.17
(g/100 kg BW)				
Recommended CP intake ^a^	169.33–199	126	Not available	126
(g/100 kg BW)				

^a^ https://www.msdvetmanual.com (accessed on 11 July 2021).; Martin-Rosset, 2018. Abbreviations: DM: dry matter; DE: Digestible Energy (according to the NRC system); CP: crude protein; BW: body weight; DP: Digestible crude protein.

**Table 3:** Weight, BCS and blood metabolic parameters of jennies in two late pregnancy and maintenance fed the same diet (Diet 1).

	Pregnancy Group	Maintenance Group	RMSE
Weight	339.86 ^A^	296.25 ^B^	24.83
BCS	3.86	3.687	0.37
Glucose (mg/dL)	50.86	53.37	12.05
NEFA (μmol/L)	68.29 ^a^	37.25 ^b^	26.77
B-HBA (mg/dL)	2.39	3.53	2.52
BUN (mg/dL)	13.00	15.62	2.71
Total blood Protein (g/dL)	7.24	7.39	0.63

A,B = *p* < 0.01; a,b = *p* < 0.05; Abbreviations: BCS: body condition score; NEFA: Not Esterified Fatty Acids; B-HBA: Beta-Hydroxybutyrate; BUN: Blood urea nitrogen; RMSE: root mean square error.

**Table 4:** Weight, BCS and blood metabolic parameters of jennies in lactation and maintenance fed the same diet (Diet 2).

	Lactating Group	Maintenance Group	RMSE
Weight	290.43	312.36	8.80
BCS	3.85 ^B^	4.22 ^A^	0.25
Glucose (mg/dL)	66.52 ^A^	55.43 ^B^	17.00
NEFA (μmol/L)	268.28 ^A^	26.37 ^B^	113.53
B-HBA (mg/dL)	3.98	3.69	1.35
BUN (mg/dL)	14.85	15.07	1.95
Total Blood Protein (g/dL)	7.15	6.88	0.37

A,B = *p* < 0.01; Abbreviations: BCS: body condition score; NEFA: Not Esterified Fatty Acids; B-HBA: Beta-Hydroxybutyrate; BUN: Blood urea nitrogen.

**Table 5:** Correlations between blood metabolic parameters and milk quality (only the statistically significant correlations are reported).

	DM (%)	Protein (%)	Casein (%)	Fat (%)	Ash (%)	pH	SCC (n/100 mL)
Glucose (mg/dL)	-	-	-	0.41 *	-	0.40 *	-
NEFA (μmol/L)	0.34 *	0.36 *	0.40 *		0.36 *		
B-HBA (mg/dL)	-	-	-	-	-	-	0.61 **
BUN (mg/dL)	-	0.36 *					
Blood total protein (g/dL)	-	-	0.39 *	-	0.38 *	-	-

* *p* < 0.05; ** *p* < 0.01. Abbreviations: DM: dry matter; SCC: somatic cell count; NEFA: Not Esterified Fatty Acids; B-HBA: Beta-Hydroxybutyrate; BUN: Blood urea nitrogen.

**Table 6:** Correlations between blood Not Esterified Fatty Acids (NEFAs) and milk fatty acid profile (only statistically significant correlations are reported).

Fatty Acid (g/100 g of Total FAME)	NEFA (μmol/L)	Fatty Acid (g/100 g of Total FAME)	NEFA (μmol/L)
C6:0	−0.57 **	C20:0	−0.46 *
C8:0	−0.55 **	C24:0	0.40 *
C10:0	−0.44 *	SCFA	−0.49 **
C12:0	−0.50 **	LCFA	0.43 *
C14:0	−0.46 *	SFA	−0.49 **
C15:0	−0.42 *	MUFA	0.60 **
C16:1	0.57 *	UFA/SFA	0.46 *
C17:0	−0.58 **		

* *p* < 0.05; ** *p* < 0.01; Abbreviations: FAME fatty acid methyl esters; SCFA: short chain fatty acids; LCFA: long chain fatty acids; SFA: saturated fatty acids; MUFA: monounsaturated fatty acids; UFA: unsaturated fatty acids.

## Data Availability

The data that support the findings of this study are available from the authors.
